# 医用内窥镜临床评价体系专家共识

**DOI:** 10.3779/j.issn.1009-3419.2020.104.10

**Published:** 2020-06-20

**Authors:** 中国医师协会胸外科医师分会, 浙江省医学会胸外科学分会, 中国胸外科肺癌联盟, 国家重点研发计划项目组

**Keywords:** 医用内窥镜, 评价指标, 专家共识, Medical endoscopy, Evaluation index, Expert consensus

## Abstract

医用内窥镜的发展速度及普及程度非常快，国际大厂品牌统领内窥镜市场半壁江山，目前国产品牌的市场规模也迅速增长，硬镜、软镜电子化技术迅速发展，逐渐接近国际先进水平。但是，目前适用于评价医用内窥镜的国家标准、行业规定等相关标准缺乏，国内外产品差距缺少定量、明确的认识，国产品牌多而杂，需要对医用内窥镜给予全面、准确的评价。在此背景下，专家组经过科学地遴选和严谨地实践提出了一套科学、系统、适用的标准化医用内窥镜临床评价体系，以期规范化各类内窥镜评价指标。

## Abstract (en)

Nowadays, there is a rising development and popularity of medical endoscopes. The international brand dominates the endoscope market. The market size of domestic brands is also growing rapidly. The electronic technologies of hard or soft endoscopes are developing rapidly and gradually approaching to the advanced level in worldwide. However, at present, there is a lack of relevant standards for national standards and industry regulations for evaluating medical endoscopes, and also lacking of quantitative and clear understanding of the gap between domestic and foreign products. It is urgently necessary to give a comprehensive and accurate evaluation to the medical endoscopes. In this context, the expert group has established a scientific, systematic, and applicable standardized medical endoscopy clinical evaluation system to standardize various endoscopic evaluation indicators.

## 1 医用内窥镜评价体系总论

近年来，国产医用内窥镜的发展速度及普及程度非常快，浙江、上海等地的医用内窥镜产业已颇具规模，市场份额也逐年增长^[1, 2]^，硬镜、软镜电子化技术迅速发展，逐渐接近国际先进水平^[3, 4]^。但是，目前国产医用内窥镜总体市场占有率仍然较低，与进口产品相比存在明显的差距^[5]^。同时，国产医用内窥镜在临床实践、可靠性、技术性能及服务体系等方面，仍有较明显的不足。

目前，国内外尚无系统性的行业标准或评价体系来评价医用内窥镜，导致各级医院临床医护人员等无法客观、合理地反馈国产与进口医用内窥镜的具体不足之处，也使国产医用内窥镜厂家无法全面、系统地认识与进口产品的差距，难以及时做出针对性的改进来提高国产医用内窥镜的竞争力。因此专家组前期已建立临床效果初级评价指标体系并设计调查问卷，再通过三轮德尔菲法完善评价指标体系，遴选出一套适用于软、硬性内窥镜的临床效果评价体系，能较充分满足内窥镜临床效果的评价需求^[6]^。

本共识旨在提供一套合理可靠的医用内窥镜临床评价指标体系，以期能真实、全面地评价国产与进口医用内窥镜的具体差异，为临床使用提供参考，为国产设备的研发、创新指出方向，推动优秀国产医用内窥镜的普及应用，促进国产医用设备的发展。

## 2 医用内窥镜评价指标

### 2.1 软性医用内窥镜评价指标

#### 2.1.1 临床效果评价指标(http://dx.doi.org/10.3779/j.issn.1009-3419.2020.104.11) 

临床效果评价指标包括客观指标与主观指标，其中客观指标包括以下：（1）关键手术步骤耗费时间：根据标准化临床场景计算关键手术步骤耗费的时间，第三方评价（第三方人员根据手术录像进行统计分析）；（2）并发症：术中发生并发症的情况，包括术中出血等，第三方评价；（3）总操作时间：手术开始至手术结束的总时间，第三方评价。

操作医师对操作性和视觉效果的主观打分并比较是国内外使用较多评价内窥镜质量及应用效果的方式^[7, 8]^，因此专家组推荐将其分为三大类主观指标进行评分。

主观指标一（视觉相关）：（1）视野：镜头显示的视野大小；（2）清晰度：画面的清晰程度；（3）成像稳定性：图像显示的稳定性及抗干扰的能力；（4）景深：可见图像的深度对操作的影响程度；（5）分辨能力：分辨组织器官细节及辨别层次的能力；（6）立体感：组织器官的显示立体感；（7）镜头抗模糊能力：术中镜头抵抗雾、烟、体液等并保持清晰的能力；（8）抗反光能力：消除术中光源引起的反光的能力；（9）图像/色彩保真性：显示图像与真实解剖结构及色彩的相似性；（10）图像总体印象：根据术中对图像的总体印象打分。

主观指标二（操作相关）：（1）镜头擦拭效果：术中因镜头模糊利用管壁等结构擦拭后重新变清晰的能力；（2）镜头擦拭便利性：术中利用管壁等结构擦拭镜头的便利程度；（3）冲洗吸引效果：术中冲洗及吸引的临床效果；（4）操作孔道通畅性：操作孔道进出相关器械的通畅程度；（5）操作精细程度：镜头、按钮等组件满足术中精细操作要求的程度；（6）目标部位显露能力：精确到达并显露目标部位的能力；（7）目标部位操作便利性：达到目标部位后进行活检等相关操作的便利性；（8）操作性总体印象：根据术中医用内窥镜总体操作性能进行打分^[9]^。

主观指标三（不适感）：手术医生不良反应（术前、术后分别评价每台手术）：（1）视疲劳：手术造成的视觉疲劳程度；（2）躯体疲劳：手术造成的躯体疲劳程度。

最终进行总体印象评分，医生根据当前使用的软性内窥镜对“临床效果”进行总体印象打分。

#### 2.1.2 临床功能及适用性评价指标(http://dx.doi.org/10.3779/j.issn.1009-3419.2020.104.12) 

临床功能及适用性评价指标分为三大类，分别是设备相关指标、图像相关指标和操作相关指标。

其中设备相关指标具体包括：（1）操作手柄交互界面：操作手柄交互界面按钮及其功能的简洁性和实用性；（2）镜柄外形：镜柄外形握持是否稳定、舒适；（3）按钮误触：常用按钮如摄像按钮等是否容易误触；（4）按钮盲操作：盲操作下是否便于进行常用功能操作如调节焦距；（5）镜头方向调节灵活性：调节镜头方向时镜柄是否灵活；（6）镜头方向稳定性：固定操作时软镜与自然腔道间阻尼能否保证镜头方向稳定；（7）镜柄重量：镜柄重量对术者操作的影响；（8）光纤长度：光纤线接头角度、长度是否容易受患者体位及操作者站位限制；（9）医用内窥镜长度：医用内窥镜长度能否满足临床要求；（10）设备可扩展性：主机与其他相关设备的兼容程度（如支气管镜与环扫超声探头及磁导航等设备是否匹配）；（11）硬度：软镜硬度大小是否合适及是否可进行改变（支持/不支持）；（12）电子染色：是否支持电子染色功能及对染色满意度（支持/不支持）；（13）副送水功能：软镜是否支持副送水进行冲洗（支持/不支持）。

图像相关指标包括：（1）高清录像：主机是否支持外接存储设备录像（支持/不支持）；（2）缩放功能：①视野放大缩小满足临床需要的能力；②如不能满足请写出期望最大倍数；（3）景深：显示解剖目标整体时是否清晰；（4）自动变焦：小范围操作移动时是否需频繁调焦；（5）高清显示：高清显示功能能否满足操作者需求。

操作相关指标包括弯曲性能：镜头弯曲角度及弯曲半径对显露目标部位的影响，如国产设备弯曲半径较大可能不能良好显示目标部位。

最终进行总体印象评分，医生根据医用内窥镜对临床功能及适用性的支持作用进行总体印象打分。

任何一项临床操作都离不开护士的技术配合，因此我们建议将护士评价纳入体系中。软性医用内窥镜护士评价部分由临床操作护士进行评价填写，指标如下。操作相关：（1）开、关机方式：腔镜系统各设备的开关机步骤是否繁琐；（2）移动灵活性：主机及组件移动位置的灵活程度；（3）腔镜拆卸及连接：腔镜系统各设备部件的拆卸、连接是否方便；（4）主机交互界面：主机交互界面按钮及其功能的简洁性和实用性；（5）清洗/消毒：腔镜系统各设备的清洗及消毒的繁琐程度。

最终护士根据医用内窥镜对临床功能及适用性的支持作用进行总体印象打分。

### 2.2 硬性医用内窥镜评价指标

#### 2.2.1 临床效果评价指标（http://dx.doi.org/10.3779/j.issn.1009-3419.2020.104.13） 

硬性医用内窥镜临床效果评价指标同样分为客观指标评价与主观指标评价。其中客观指标包括：（1）关键手术步骤耗费时间：根据标准化临床场景计算关键手术步骤耗费的时间，第三方评价（第三方人员根据手术录像进行统计分析）；（2）关键手术步骤操作错误的次数：关键手术步骤操作错误的次数，第三方评价；（3）并发症：术中发生并发症的情况，包括术中出血等，第三方评价；（4）总操作时间：手术开始至手术结束的总时间，第三方评价；（5）中转开放：腔镜中转开放，腔镜无法满足手术操作的需要必须进行开放手术，第三方评价。主观指标：主要是操作医生对医用内窥镜系统相对主观的评价。

主观指标是操作医生对医用内窥镜系统相对主观的评价，专家组推荐将其分为两大类进行评价。

主观指标一（视觉相关）：（1）视野：镜头显示的视野大小；（2）清晰度：画面的清晰程度；（3）成像稳定性：图像显示的稳定性及抗干扰的能力；（4）景深：可见图像的深度对操作的影响程度；（5）分辨能力：分辨组织器官细节及辨别层次的能力；（6）对比度：明暗区域的差异程度；（7）立体感：组织器官的显示立体感；（8）镜头抗烟雾能力：镜头对术中雾/烟的抵抗能力；（9）抗反光能力：消除术中光源引起的反光的能力；（10）图像/色彩保真性：显示图像与真实解剖结构及色彩的相似性；（11）图像总体印象：根据术中对图像的总体印象打分；（12）变焦能力：对焦的及时性和准确性，是否能自动调焦或是否需要手动调焦。

主观指标二（不适感）：（1）视疲劳：手术造成的视觉疲劳程度；（2）躯体疲劳：手术造成的躯体疲劳程度。

最终进行总体印象评分，操作医师将根据当前使用的硬性内窥镜对“临床效果”进行总体印象打分。

#### 2.2.2 临床功能及适用性评价指标（http://dx.doi.org/10.3779/j.issn.1009-3419.2020.104.14） 

在对硬性内窥镜进行临床功能及适用性评价时，专家组推荐将其分为两大类指标进行评价，分别是设备相关指标和图像相关指标。

设备相关指标：（1）操作手柄交互界面：操作手柄交互界面按钮及其功能的简洁性和实用性；（2）镜柄外形：镜柄外形握持是否稳定、舒适；按钮误触：（3）常用按钮如摄像按钮等是否容易误触；（4）按钮盲操作：盲操作下是否便于进行常用功能操作如调节焦距；（5）镜头方向调节灵活性：调节镜头方向是否灵活；（6）镜头方向稳定性：固定操作时方向是否稳定；（7）内镜光纤：光纤线接头角度、长度是否容易受患者体位及术者站位限制；（8）镜柄重量：镜柄重量对术者操作的影响；（9）医用内窥镜长度：医用内窥镜长度是否满足临床要求；（10）发热程度：镜柄发热造成的不适感^[10]^。

图像相关指标：（1）自体荧光成像：是否具备自体荧光成像及其性能（支持/不支持）；（2）缩放功能：视野放大缩小满足临床需要的能力，如不能满足请写出期望倍数；（3）高清显示及录像功能：是否能显示高清图像并录像；（4）景深：显示解剖目标整体时是否清晰；（5）自动变焦：小范围操作移动时是否会频繁调焦；（6）镜头抗污/雾功能：镜头抗污/雾功能。

最终进行总体印象评分，医生根据医用硬性内窥镜对临床功能及适用性的支持作用进行总体印象打分。

硬性医用内窥镜护士评价部分由临床操作护士进行评价填写。操作相关指标包括：（1）开、关机方式：腔镜系统各设备的开关机步骤是否繁琐；（2）移动灵活性：主机及组件移动位置的灵活程度；（3）腔镜拆卸及连接：腔镜系统各设备部件的拆卸、连接是否方便；（4）主机交互界面：主机交互界面按钮及其功能的简洁性和实用性；（5）清洗/消毒：腔镜系统各设备的清洗及消毒的繁琐程度。

最终护士根据医用内窥镜对临床功能及适用性的支持作用进行总体印象打分。

## 3 医用内窥镜评价标准化临床评价场景及人员

### 3.1 软性医用内窥镜

专家组建议以纤维支气管镜常规检查为例进行规范：（1）操作医师要有资质要求（三甲医院要求中级职称或以上，基层医院要求高级职称，所有操作者需熟练掌握本专业医用内窥镜各项操作），使用模型训练器设计标准化操作流程，对参加课题的医生进行操作测试，通过测试的医生被认定其技能符合本课题设计要求。所有临床医师需要接受眼科的检查，包括视敏度、颜色分辨力、视力、立体感等，检查合格者纳入评测人员。（2）患者评估：收集临床基本信息（患者一般信息、病灶部位、大小、诊断等），操作室的布局在研究期间尽量保持一致，包括照明条件、操作团队的站位以及医用内窥镜系统、操作台等摆放。（3）操作设立分级（设立监督顾问，并根据以下原则分级）：1级（鼻腔通畅；声门麻醉效果好；没有明显的支气管畸形；患者配合佳），2级（鼻腔狭小患者；声门稍活跃；患者配合一般），3级（声门活跃；解剖结构异常；患者配合差）。（4）关键操作步骤分解，任务1：支气管镜经鼻/口腔到达声门，计时从支气管镜进入鼻腔开始，到达会厌后方显露声门。任务2：进入声门，计时从尝试进入声门到成功进入气管内为止。任务3：检查，计时从显露隆突到最终检查部位。任务4：支气管肺泡灌洗，计时从经操作孔注射生理盐水开始到灌洗结束。任务5：支气管黏膜刷检，计时从经操作孔插入毛刷开始到刷检结束。

### 3.2 硬性医用内窥镜

专家组建议以胸腔镜肺叶切除术为例进行规范：（1）手术、操作医师要有资质要求（三甲医院要求中级职称或以上，基层医院要求高级职称，所有操作者需熟练掌握本专业医用内窥镜各项操作），使用腔镜模拟训练器设计标准化操作流程，对参加课题的手术医生进行操作测试，通过测试的医生被认定其手术技能符合评测要求。所有临床医师需要接受眼科的检查，包括视敏度、颜色分辨力、视力、立体感等，检查合格者纳入评测人员。（2）患者评估，知情同意，收集临床基本信息（患者一般信息及疾病基本信息，可在移动端数据采集平台拍照上传病历及手术记录），手术室的布局在研究期间尽量保持一致，包括照明条件（在腔镜操作过程中，无影灯应处于关闭状态）、手术团队的站位以及医用内窥镜系统、手术台等。（3）手术首先进行胸腔探查明确手术困难程度^[11]^，并设立手术分级（设立监督顾问，并根据以下原则分级）：1级（胸腔、肺门周围没有粘连；牵拉肺叶后肺静脉可良好显示；没有明显的血管、支气管畸形；叶间裂发育完好），2级（肥胖/胸腔狭小患者；肺门周围富含脂肪；肺门膜状、疏松粘连；支气管周围淋巴结稍肿大；叶间裂发育一般），3级（肺门周围致密粘连；全胸腔粘连；解剖结构困难、异常、模糊；支气管周围淋巴结肿大、钙化明显等；叶间裂发育差）；（4）关键手术步骤分解^[12, 13]^，任务1：肺门区解剖肺静脉，计时从抓好肺叶牵拉开始，到肺静脉夹闭离断。任务2：肺门区解剖肺动脉，计时从肺动脉游离到夹闭离断。任务3：肺叶支气管夹闭离断，计时从肺叶支气管游离到夹闭离断。任务4：叶间裂离断，计时从叶间裂切割离断开始到肺叶完整游离下来。

## 4 医用内窥镜评价标准化数据采集及处理

专家组建议采用5级打分模式进行各指标的量化。1分代表无法操作；2分代表严重影响操作；3分代表操作体验不佳；4分代表正常操作；5分代表操作非常舒适。我们建议开发或者使用专家团推荐的移动端数据采集平台——内镜评测，标准化评测数据的采集与录入，尽可能减少数据采集过程中人为及主观因素导致的误差，及时客观地记录及保存评测数据，并上传到云端供下一步数据处理分析，最终我们建议统计学专家对采集的数据进行清洗、甄别后，再利用科学、有效的统计学方法分析数据。

综上所述，科学、系统、适用的医用内窥镜临床评价标准化体系需要从临床真实世界情况出发^[14]^，所建立的评价指标要能充分涵盖临床使用中的方方面面，满足医用内窥镜临床评价的需求，力求真实、全面地评价国产与进口医用内窥镜的差异，为国产医用内窥镜的研发、创新指出方向，促进国产设备的改进和技术提升，提升其国际竞争力。
